# Dosage Effect of Wet-Process Tuff Silt Powder as an Alternative Material of Sand on the Performance of Reactive Powder Concrete

**DOI:** 10.3390/ma15113930

**Published:** 2022-05-31

**Authors:** Yanxia Cai, Zhi Lin, Jingrui Zhang, Kaiji Lu, Linbing Wang, Yue Zhao, Qianlong Huang

**Affiliations:** 1 Beijing Zhonglu Gaoke Highway Technology Co., Ltd., Beijing 100088, China; 13311530926@163.com (Y.C.); kj.lu@rioh.cn (K.L.); 2 Research and Development Center of Transport Industry of New Materials, Technologies Application for Highway Construction and Maintenance, Beijing 100088, China; 3 Research Institute of Highway Ministry of Transport, Beijing 100088, China; 4 Zhejiang Comm Mining Co., Ltd., Zhoushan 316000, China; nblinzhi3291@163.com; 5 School of Civil Engineering, Hebei University of Engineering, Handan 056038, China; 6 Department Civil & Environmental Engineering, Virginia Tech, Blacksburg, VA 24061, USA; wangl@vt.edu; 7 Beijing General Research Institute of Mining and Metallurgy, Beijing 100160, China; zhaoyue@bgrimm.com

**Keywords:** wet-process tuff silt powder, RPC, mechanical properties, microstructure

## Abstract

A large amount of stone powder is produced during the production of machine-made sand. This research aims to study the effect of wet-process tuff silt powder (WTSP) dosages (as an alternative sand material to utilize waste stone powder and reduce environmental hazards) on reactive powder concrete’s (RPC) mechanical performance. The physical and chemical properties of WTSP were analyzed as per relevant standards. This study prepared RPC samples with various WTSP content (0%, 6%, 12%, and 18%) to replace quartz sand at the same water–binder ratio (0.14) and allowed the samples to cure for 3 days, 7 days and 28 days prior to unconfined compression testing and flexural testing. Scanning electron microscopy (SEM) and Mercury Intrusion Porosimetry (MIP) testing were also carried out to observe the evolution of macroscopic properties in response to replacing part of quartz sand with the same amount of WTSP. The results show that the developed flexural and unconfined compressive strength (UCS) decreases slowly with a greater dosage of WTSP. However, when the WTSP content is 12% or less, the RPC made with WTSP satisfies the industrial application threshold regarding mechanical properties. For RPC samples containing more than 12% WTSP, the UCS and flexural strength showed a dramatic drop. Thus 12% of WTSP content was deemed the maximum and the corresponding UCS of 104.6 MPa and flexural strength of 12 MPa for 28 days of curing were the optimums. The microscopic characteristics indicate that the addition of WTSP can effectively fill the large pores in the RPC micro-structure, hence reducing the porosity of RPC. Furthermore, the WTSP can react with the cementitious material to form calcium aluminate during the hydration process, further strengthening the interface. The alkaline calcium carbonate in WTSP could improve the interfacial adhesion and make the structure stronger.

## 1. Introduction

Sand and gravel are considered one of the most consumed non-renewable natural resources, which are vital to many industries, especially in building and road construction [1,2]. Since 2018, up to 50 billion tonnes of sand and gravel have been mined each year globally, leading to riverbank collapse, sinking deltas, coastal erosion, and biodiversity loss and causing environmental damage worldwide [3]. In recent years, natural sand and gravel have been gradually depleting with large-scale exploitation [4]. With the urbanization of many developing countries (especially China), the sand and gravel production has risen to a high level, and the price of sand and gravel has increased dramatically over the last decade, more than ten times in some areas, due to the shortage of sand and gravel [5]. In addition, with the awareness of sustainable development, to protect the natural ecological environment, various regions have introduced policies to restrict or ban natural sand mining [6,7,8,9]. As an alternative resource to natural sand, machine-made sand, obtained from crushing larger aggregate pieces into sand-sized aggregate particles, has been promoted and widely used in recent decades [10,11,12].

In 2020, China’s total machine-made sand and gravel production was 19 billion tons [4,13,14,15]. According to processes, there are three main machine-made aggregate production methods: wet production, dry production, and dry–wet combination production. In Zhejiang, China, one artificial sand company used the wet method for machine-made sand production as the wet production method has the characteristics of a clean aggregate surface and low environmental pollution [16]. Waste material, called stone powder, is obtained from crushing larger aggregate pieces during sand production. In general, stone powder production accounts for about 10% of the total machine-made sand generation.

The accumulated waste material (stone powder) leads to serious environmental issues and requires a large number of land resources to deposit waste stone powder [17]. The utilization and treatment of stone powder have become a massive challenge for the sustainable development of China’s building industry.

Reactive powder concrete (RPC) is a new type of cement-based composite material developed by French scholar Richard et al., in 1993 [18,19]. Its basic preparation principle is based on the closest packing theory, removing coarse aggregate, taking fine quartz sand as aggregate, and adding a high-efficiency water reducer and an appropriate amount of steel fiber. The main raw materials also include fine mineral admixtures such as silica fume and fly ash, Thus, internal defects are reduced to improve the compressive strength, toughness and durability of concrete [20,21]. The cover plate made of RPC has the characteristics of high strength, high durability, lightweight, easy modeling, high service life and remarkable long-term benefits but its cost is high. At present, RPC products such as RPC sidewalk cover plates and RPC cable trench cover plates are not widely used.

Studies have shown that stone powder can be used as an auxiliary cementing material or fine aggregate to replace part of the cement or fine aggregate to enhance the performance of concrete. Chadli Mounira et al. [22] added 30% of granite waste stone powder to reactive powder concrete (RPC) to replace quartz sand and improve RPC’s mechanical properties. Fatih Hattatoglu et al. [23] used ahlat stone powder (ASP) as ignimbrite powder instead of cement in the production of RPC. The binder percentages obtaining the highest compressive and flexural strength were determined by changing the amounts of cement, so as to reduce the cost of the RPC. Yu et al. [24] replaced machine-made sand with granite porphyry powder to improve concrete’s mechanical properties and durability. Jiang and Wang [25] illustrated that using natural river sand as aggregate and stone powder together as micro-aggregate to prepare RPC is feasible. The addition of stone powder can reduce the pore size and porosity of RPC. Fan [26] explored the feasibility of using marble powder and granite powder as cement admixtures and found that adding stone powder and granite powder would shorten the setting time of cement. When the amount of marble powder does not exceed 10%, it is beneficial to improve the fluidity of cement mortar. The contribution rate of marble powder to the strength of cement mortar is higher than that of granite powder, and the tuff rock powder is not used as a stone powder for concrete. Yang et al. [27] replaced fly ash with stone powder and studied the workability and compressive strength of concrete, which provided a certain reference for the rational use of stone powder in concrete. Yuan [28] conducted experiments to evaluate the stone powder content effects on concrete compressive strength and flexural strength, slump, chloride ion diffusion coefficient, and freeze–thaw resistance. The results show that when the content of stone powder is 5~10%, adding stone powder can effectively improve compressive strength, flexural strength, slump, freeze–thaw resistance, and other mechanical properties of concrete.

Currently, the research on stone powder mainly focuses on applying granite stone powder, limestone powder, marble powder and other wastes as mineral admixtures or replacing parts of cement and sand in concrete [29]. However, there are few studies on the wet-processed tuff silt powder (WTSP) and the application of WTSP in high-performance concrete. The addition of WTSP partially replacing quartz sand in RPC can realize the resource utilization of waste.

In this paper, the effects of WTSP as an alternative material of sand on the mechanical properties and microstructures of RPC were studied. The WTSP is used to replace part of quartz sand in RPC, which reduces the amount of quartz sand, hence reducing the RPC’s preparation cost and promoting the resource utilization of waste stone powder. It is also of great significance for alleviating environmental pollution and provides a reference basis for applying stone powder in RPC.

## 2. Material

### 2.1. Cementitious Material

Ordinary Portland Cement (OPC 42.5) from a local manufacturer was used as the cementitious material. The physical properties and chemical composition of the cement, determined as per relevant standards are shown in Table A1 and Table A2 in the Appendix A. This OPC has a density of 3.12 g/cm^3^ and a specific surface area of 355 m^2^/kg. The measured 28-day standard Flexural and Compressive strengths of the cement paste were 9.3 MPa and 53.2 MPa, respectively. The chemical composition of OPC mainly consists of carbon oxide (CaO) and silicon dioxide (SiO_2_), with mass fractions of 63.57% and 20.58%, respectively.

Silica fume, microbeads powder, and mineral filler from the same manufacturer were used as additional cementitious material to prepare reactive powder concrete. Their physical and chemical properties are illustrated in Table A3, Table A4 and Table A5 in the Appendix A. The silica fume is SF93 type silica fume, where the SiO_2_ content is 94.61%, and the specific surface area is 18,648 m^2^/kg. The microbeads powder adopts an uffa2.0-type microbeads powder; mineral powder adopts an S95-type mineral powder with a density of 2.9 g/cm^3^.

### 2.2. Aggregate

Quartz sand from a local manufacturer was used as the original aggregate material, which will be partially replaced by WTSP. The chemical composition of the quartz sand is summarised in Table A6 in the Appendix A, which indicates that the quartz sand consists of 98.21% of silicon dioxide (SiO_2_). The particle size distribution shows that more than 90% of quartz sand is retained between 0.85 and 0.425 mm sieves, and the quartz sand’s fineness modulus (FM) is 3.11.

**Figure 1:**
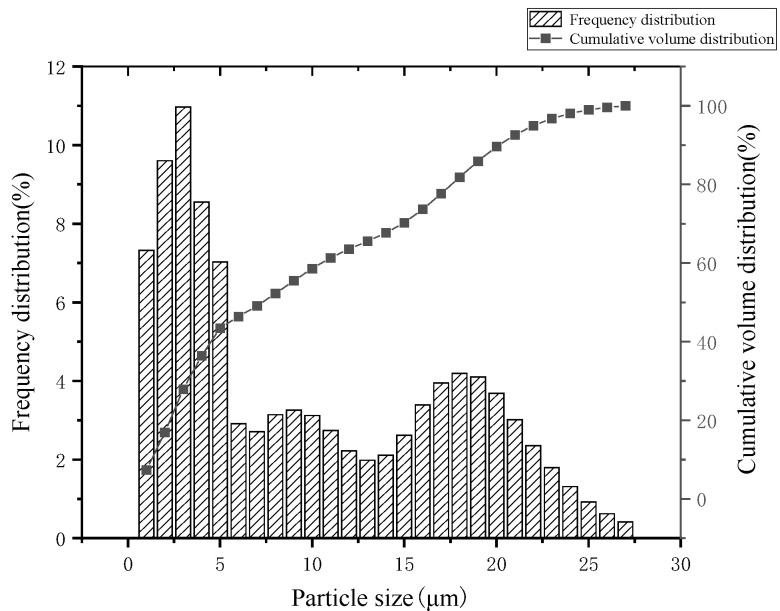
The frequency division and cumulative volume distribution of WTSP particle size.

A manufactured WTSP from Zhejiang Comm Mining Co., Ltd., Zhoushan, China, wet-collected during mining production, was used as an alternative aggregate material to replace quartz sand. Its properties are shown in Table A7 in the Appendix A. The particle size distribution of the WTSP was measured by Malvern Mastersizer 2000 (Malvern Instruments Ltd., Malvern, UK) and displayed in Figure 1.

An X-ray fluorescence test (XRF) and X-ray diffraction test (XRD) were used for the chemical and mineralogical analysis of the WTSP. As shown in Table A8 in the Appendix A, the mineralogical composition of the stone power consists of feldspar (45.9%), quartz (20.6%), kaolinite (14.5%), montmorillonite (7.7%), calcite (5.9%), illite (4%), chlorite (1.1%) and pyrite (0.2%). As shown in Table A9 in the Appendix A, the WTSP’s chemical composition mainly consists of silicon dioxide (SiO_2_), Aluminium oxide (Al_2_O_3_), Potassium oxide (K_2_O), and ferric oxide(Fe_2_O_3_), with mass fractions of 61.51%, 16.40%, 4.91% and 4.28%, respectively; The corrosion analysis results in Table A10 in the Appendix A indicate the stone power has a pH value of 8.95 and contains HCO_3_^−^, Ca^2+^, Cl^−^, SO_4_^2−^.

From Figure 1 and Table A7, Table A8, Table A9 and Table A10 in the Appendix A, it can be seen that the average particle size of WTSP is 9.38 μm, and the small particle size helps to achieve compact filling of concrete and increase strength. The main crystalline minerals are quartz, feldspar, and kaolinite, which account for 81% of the total phase composition, and the main chemical components are SiO_2_ and Al_2_O_3_, with their content up to 77.92%; through the analysis of corrosive substances, the pH of the WTSP is weakly alkaline. The content of corrosive substances is low. The compressive strength ratio of 7 days and 28 days shows that the WTSP has a certain activity, indicating that it is feasible to use WTSP to prepare reactive powder concrete.

### 2.3. Additives, Fiber, and the Mixing Water

The copper-plated flat steel fiber with a length of 13 mm, a diameter of 0.18 mm, and tensile strength of 3105 MPa was used to improve the strength of RPC.

A salt high-performance water reducing agent (Polycarboxylate superplasticizer), produced by Sika (China) Co., Ltd., Suzhou, China, was used to maintain the flowability of mixed slurry and the water-reducing efficiency of the agent is 33%.

Tap water was used for the slurry preparations.

## 3. Methodology

### 3.1. Mix Designs

To study the dosage influence of WTSP as an alternative sand material on reactive powder concrete performance, a total of 5 mix designs, as outlined in Table 1, were examined. Various stone power contents (0%, 6%, 12%, and 18%) were used to replace quartz sand at the same water–binder ratio (0.14). Sample A-0 prepared with 0% WTSP replacement was used as a reference specimen, which provides the standard unconfined compressive strength and flexural strength of RPC.

The dosage of the water reducing agent was used to adjust the fluidity of various mixed slurries; the fluidity of all RPC slurries should be greater than 180 mm. The amount of water-reducing agent needed shows an increasing trend with higher WTSP dosage. This is because the average particle size of WTSP and quartz sand of the same quality is much smaller than that of quartz sand. The surface area and the water demand ratio are large to achieve the same fluidity effect, which leads to an increase in the amount of water-reducing agent.

As illustrated in Table 1, an increasing WTSP content leads to a higher water-reducing agent requirement. The dosage of the water reducing agent is adjusted with the fluidity as the reference, and the fluidity of RPC is between 180–200 mm.

### 3.2. Mixing, Forming and Curing

The designed amount of aggregates (quartz sand, WTSP), steel fibers, and cementitious materials (cement, silica fume, mineral powder, fly ash) were blended in dry form in accordance with the selected mix designs outlined in Table 1. First, the dry materials were mixed for 4 min. The required amount of water and admixtures (water-reducing agent, defoaming agent, desulfurization gypsum) was added to the mixer and mixed for approximately 8 min to obtain slurries of uniform consistency. The prepared mixed slurry was poured into the test molds (two types of molds were used in this study, cubic molds with a dimension of 100 mm × 100 mm × 100 mm for UCS, prism molds with a dimension of 40 mm × 40 mm × 160 mm for flexural strength) and then vibrated on the vibrating table for 2 min to remove any entrapped air.

After 24 h of curing at atmosphere conditions, the specimens were demolded and moved into the accelerated curing box for curing, with an ambient temperature increased from 25 °C to 75 °C, at a heating rate of 10 °C/hour, and the temperature is kept at 75 °C for 24 h. After that, the specimens were naturally cooled in the curing box for 24 h and cured in atmosphere condition for designed dates (3 days, 7 days and 28 days).

### 3.3. Test Method

#### 3.3.1. Unconfined Compressive Strength

**Figure 2:**
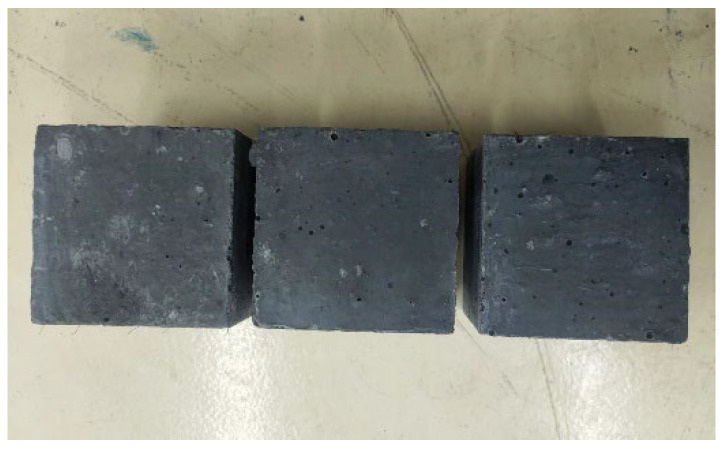
Cubic samples for unconfined compressive strength tests.

**Figure 3:**
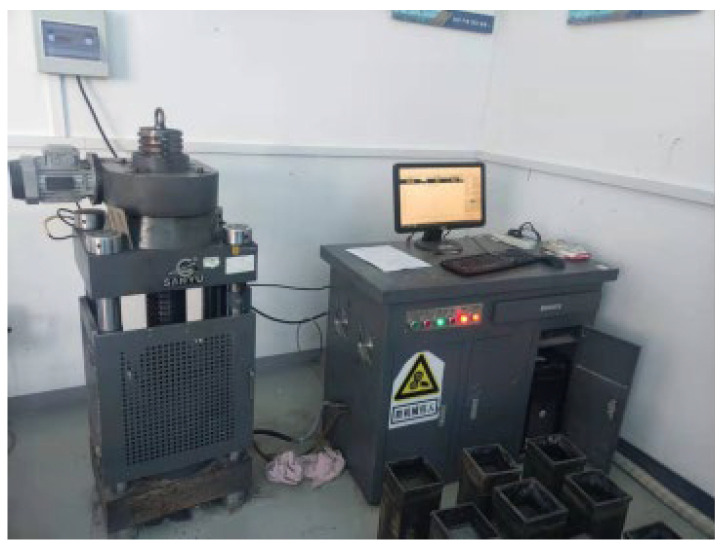
Electro-hydraulic servo pressure testing machine.

Unconfined compressive strength tests were conducted for 100 mm × 100 mm × 100 mm cubic samples (see Figure 2) at designed curing periods using a Micro-electro-hydraulic servo pressure testing machine (HYE-2000, Hebei Sanyu Weiye Testing Machine Co., Ltd., Beijing, China) with a maximum capacity of 2000 kN (see Figure 3) at 1.2 MPa/s loading rate, in accordance with the Standards for Test Methods of Physical and Mechanical Properties of Concrete (GB/T 50081–2019). The number of cube samples at each predetermined curing period is 3.

#### 3.3.2. Flexural Strength

**Figure 4:**
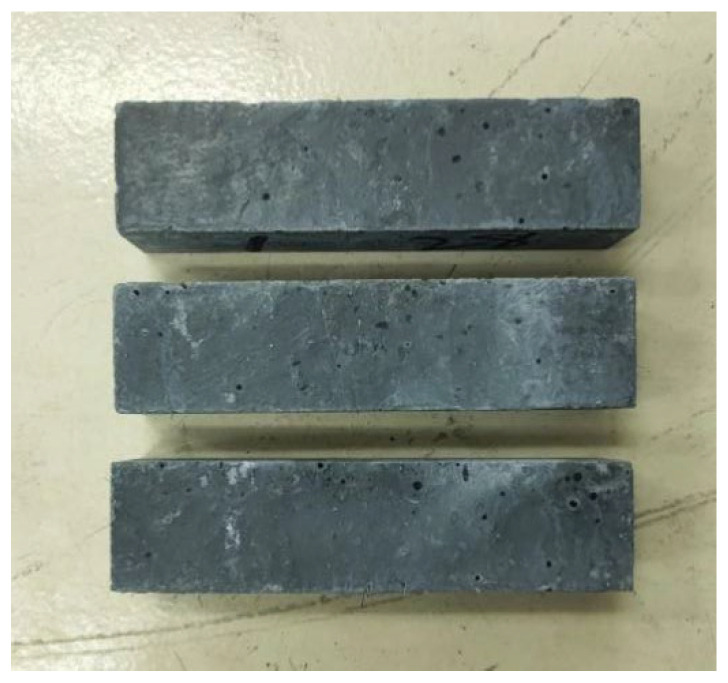
Prism samples for flexural strength tests.

**Figure 5:**
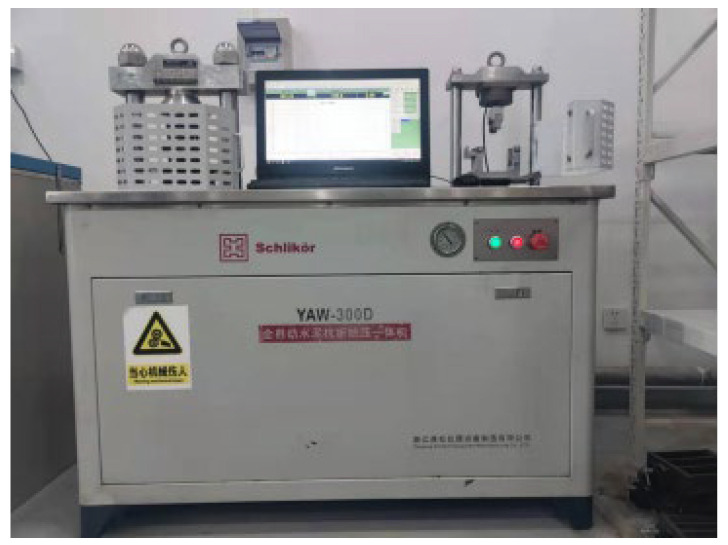
Fully automatic concrete flexural compressive all-in-one.

A 40 mm × 40 mm × 160 mm prism sample (see Figure 4) was used for the flexural strength test (FS). The flexural strength of samples at the predetermined curing periods (3 days, 7 days, and 28 days) was determined via a fully automatic concrete flexural, compressive loading machine (YAW-300D, Zhejiang Schlikor equipment manufacturing Co., Ltd., Shaoxing, China), according to the Standards for Test Methods of Physical and Mechanical Properties of Concrete (GB/T 50081-2019), with a maximum capacity of 2000 kN (see Figure 5), at a load loading rate of 0.08 MPa/s. The number of beam samples at each predetermined curing period is 3.

#### 3.3.3. Microstructure

**Figure 6:**
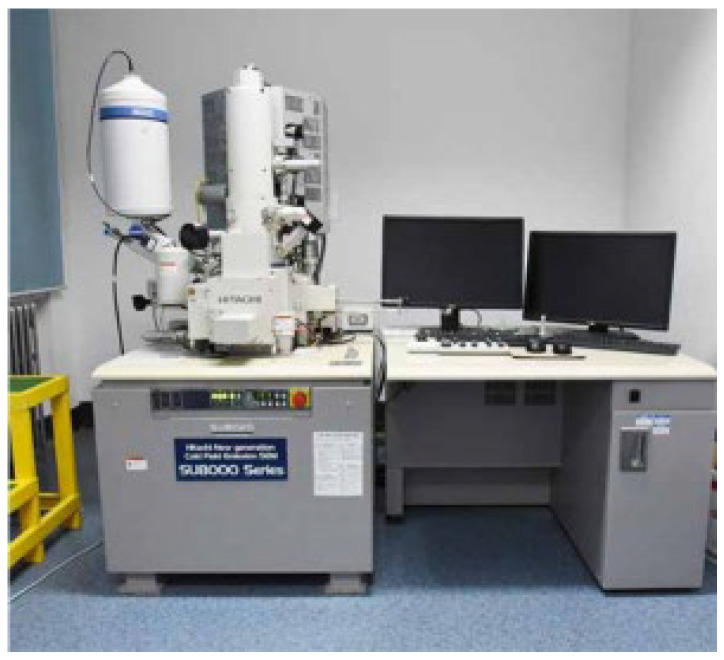
Field emission electron microscope.

Scanning Electron Microscopy (SEM) is an observational means between transmission electron microscopy and optical microscopy. It uses a narrow-focused high-energy electron beam to scan the sample, acquire various physical information through the interaction between the beam and the material, and collect, amplify, and re-image the information to characterize the microscopic morphology of the material. The microscopic morphology of the samples cured for 3 days and 28 days were observed with a Japanese electron S4800 scanning electron microscope (see Figure 6).

Mercury intrusion porosimetry (MIP) was conducted to understand the pore volume of the corresponding pore size by measuring the amount of mercury entering the pores under different external pressures. The pore structure of the samples cured for 28 days was analyzed by a high-performance automatic mercury porosimeter AutoPore V9620 (Software Version 2.03.00, Micromeritics Instrument Corporation, Atlanta, GA, USA) produced by McMerritik, USA. The differential curves of porosity and pore size distribution were automatically analyzed and recorded.

## 4. Analysis of Test Results

### 4.1. Unconfined Compressive Strength of RPC

**Figure 7:**
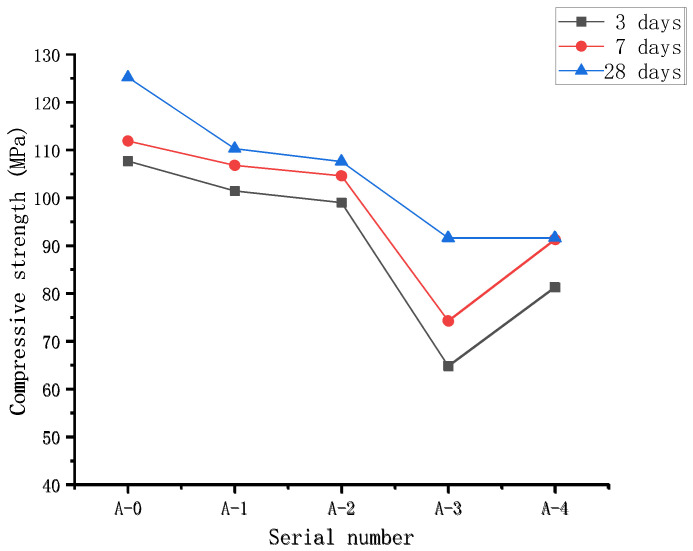
The unconfined compressive strength of RPC with WTSP replacing part of quartz sand.

The UCS result of RPC samples prepared with various WTSP contents to replace quartz sand is shown in Figure 7. Overall, the compressive strength of RPC gradually increases with the increase in the curing period. With the increase in WTSP content, the compressive strength of RPC showed a downward trend. By adding 2% desulfurized gypsum to the A-4 sample, the compressive strength of 3 days and 7 days increased significantly, and so did the compressive strength of 28 days. The increase in strength is not apparent. This is because the SO_3_ content in RPC is increased after adding desulfurized gypsum. Its stimulating effect and crystallization effect on sulfate activity will further improve the pozzolanic, morphological, and filling effect of other admixtures, thereby increasing the RPC mechanical properties [30]. A certain amount of desulfurized gypsum can increase the RPC’s early strength and slightly improve the RPC’s long-term strength [31,32,33]. The 28-day compressive strength of sample A-1 is 110.3 MPa, 11.9% lower than that of the reference sample A-0. The 28-day compressive strength of sample A-2 is 104.6 MPa, 14.1% lower than that of reference sample A-0. The 28-day compressive strength of sample A-3 is 91.6 MPa, 26.8% lower than that of reference sample A-0.

The downward trend of RPC strength with the increase in WTSP content is mainly due to the low water content in RPC slurries. The used water-reducing agent maintains the fluidity without adding water for pumping purposes. However, the WTSP has a large specific surface area. It absorbs water and leads to insufficient water for the hydration process of cementitious material, the dispersion of WTSP within the cement matrix is poor [34,35], decreasing the UCS of RPC [36,37]. In addition, there are certain impurities in the WTSP, and the mud content is significant. With the increase in the WTSP content, the increasing amount of impurities and mud in WTSP might decelerate the hydration process in RPC [38].

On the other hand, the WTSP has a lower silicon–alumina oxide content than the quartz sand, and the strength of WTSP is lower than that of quartz sand, leading to a total lower strength. By replacing quartz sand with WTSP, the aggregate gradation of the prepared mixture was changed, hence influencing the development of RPC compressive strength.

### 4.2. Flexural Strength

**Figure 8:**
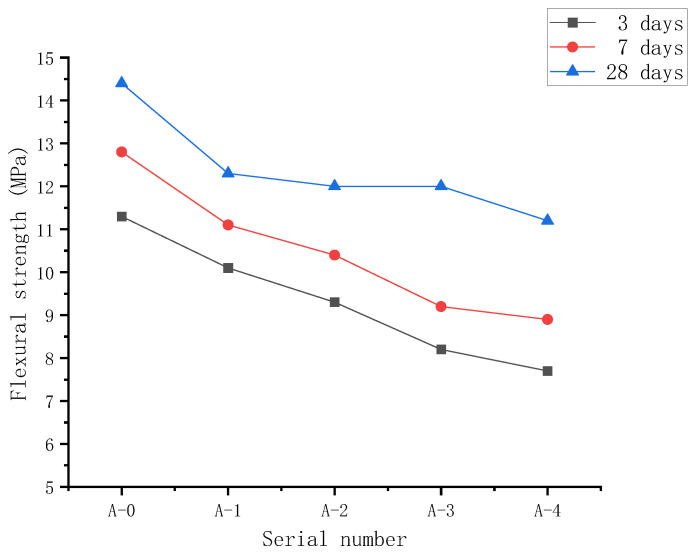
The flexural strength of RPC with WTSP replacing part of quartz sand.

Figure 8 illustrates the flexural strength of RPC samples prepared with various WTSP contents to replace quartz sand. In general, for samples at the curing period of 3 days, 7 days, and 28 days, the flexural strength decreased with the increasing amount of wet-processed WTSP. The cement hydration reaction is delayed due to the increase in WTSP replacement and the increasing amount of water-reducing agent. Furthermore, the addition of 2% desulfurized gypsum, sample A-4, shows a retarding effect on the binder hydration, and the setting time is 1.6 times that of the control group, which further affects the flexural strength of RPC [39]. When the curing age is 28 days, the flexural strength of sample A-1 is significantly lower than the flexural strength of the reference group by 14.6%, and the flexural strength is 12.3 MPa.

With the increase in WTSP content, the flexural strength further decreases and was found rather marginal. Compared with the flexural strength of the reference group, the flexural strength of sample A–2 and sample A–3 decreased by 16.7% and 18.1%, and the flexural strength was 12.0 MPa and 11.8 MPa, respectively.

Replacing part of the quartz sand with WTSP will adversely affect the flexural strength of RPC. The downward trend of flexural strength is due to the particular amount of mud in the WTSP. With the increase in the amount of WTSP, the mud content also increases, which affects the hydration of cement. On the other hand, the Zeta potential of WTSP is greater than that of cement, and the adsorption rate of water reducer is higher than that of cement, which leads to the increase in the amount of water reducer [6], the increase in the amount of water-reducing agent affects the RPC flexural strength as well. It can be found from Figure 8 that the replacement of part of quartz sand by WTSP has an effect on the flexural strength of RPC. This is due to the active SiO_2_ and Al_2_O_3_ components in WTSP. It could react with the cement hydration product Ca (OH)_2_ to generate more Calcium Silicate Hydrate (C-S-H) gel, this is conducive to the development of the flexural strength of RPC. In addition, the WTSP contains a large amount of rough and fine particles, which changes the gradation of aggregates and improves the cohesion between particles and the section compactness. The improved cohesion between particles and good cross-sectional compactness have certain positive effects on enhancing the flexural strength of concrete [40,41].

### 4.3. Analysis of Micro Characteristics

**Figure 9:**
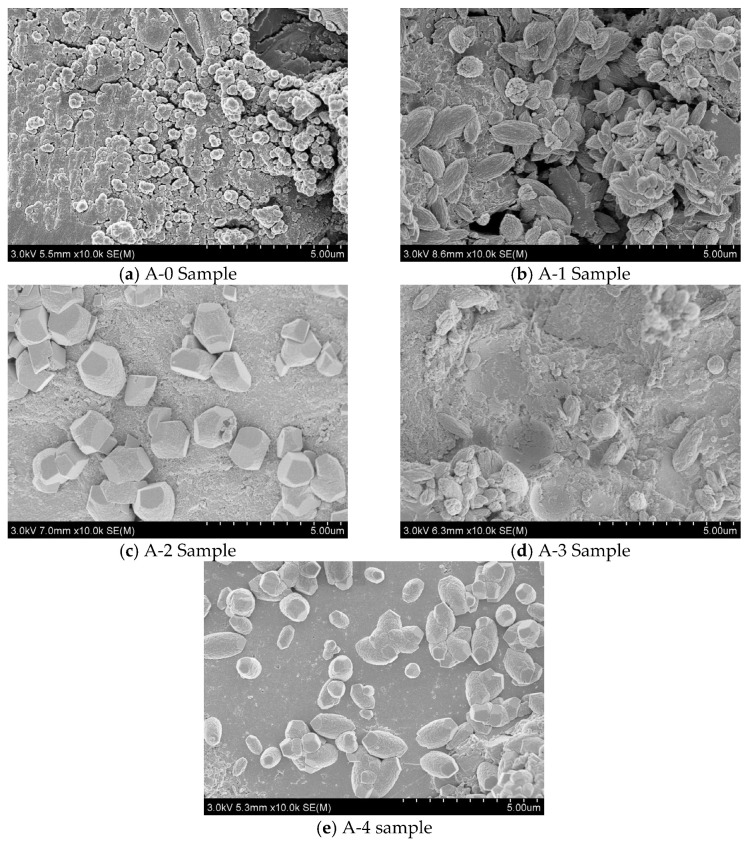
Three-day SEM photo of RPC sample.

**Figure 10:**
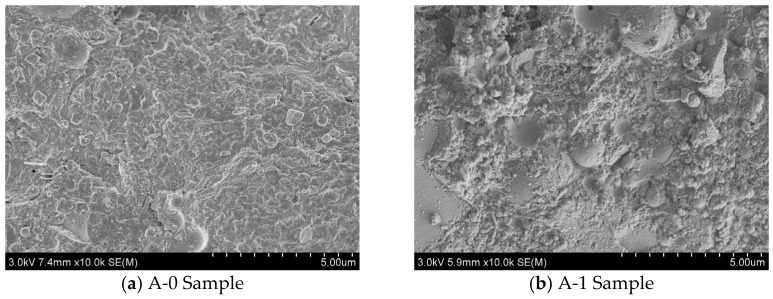

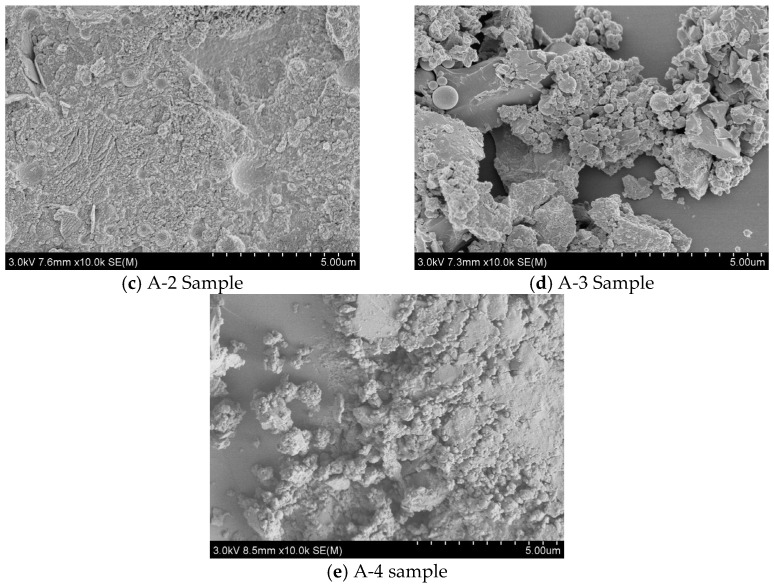
Twenty-eighth day SEM photo of RPC sample.

In order to further study the influence of WTSP dosage on the mechanical properties of RPC, the specimens with various mix designs at the age of 3 days and 28 days were analyzed by scanning electron microscopy (SEM). Figure 9 and Figure 10 illustrate the SEM micrographs for the samples A-0 to A-4 cured for 3 days and 28 days, respectively.

Figure 9a shows that the SEM micrograph for reference sample A-0 has obvious cracks and pores with a loose structure, and exposed quartz sand could be observed. Figure 9b shows that a small amount of spindle-shaped Monosulfur calcium sulfoaluminate hydrate (AFm) crystals could be observed in A-1 samples after 3 days of hydration. However, a small amount of delicate pores and some un-hydrated Ca (OH)_2_ crystals are also distributed on the micrograph. When the WTSP content is 12%, as shown in Figure 9c, the SEM micrographs of sample A-2 contain hexagonal plate-shaped calcium trisulfide hydrated calcium sulfoaluminate (AFt) crystals. When the WTSP content is 18%, in Figure 9d, a small amount of Ca (OH)_2_ crystals are found in the micrograph of sample A-3 and a dense microstructure could be found on the surface of sample A-3. After adding 2% desulfurized gypsum, in Figure 9e, the hydration produced rod-shaped AFt crystals could be observed. Overall, in Figure 9, for all RPC samples cured for 3 days, the SEM micrographs all showed un-hydrated substances, which indicates the uncompleted cement hydration process at an early age and influences the overall UCS and flexural strength. In Figure 10a, the structure of the interface zone of the A-0 sample is loose and lamellar Ca(OH)_2_ crystals are locally layered. The structural morphology of the interface area between the A-1 sample in Figure 10b and the A-4 sample in Figure 10e is obviously loose, and the exposed quartz sand particles can also be seen.

In Figure 10c, there are a few granular and lamellar Ca (OH)_2_ crystals in the interface area of the A-2 sample, and the interface area is dense. Figure 10d shows rod-shaped C-S-H and a small amount of granular Ca (OH)_2_ crystal interface area in the A-3 sample interface area. The results show that WTSP has a nucleation effect, due to the small size and high surface energy of WTSP, this enhances the nucleation effect and the deposition of hydration products on its surface so as to form a denser microstructure [42,43,44]. The WTSP has a pozzolanic effect, it can chemically react with tricalcium aluminate (C_3_A) and tetracalcium ferric aluminate (C_4_AF) in the cement during cement hydration to form hydrated calcium aluminate. The formation of alkaline calcium carbonate in wet-processed WTSP makes the hydration products tricalcium silicate (C_3_S) and CaCO_3_ have a denser micro-interface structure, while Ca (OH)_2_ forms crystals on the surface of CaCO_3_, which makes the Ca (OH)_2_ grains refined, thereby improving the interfacial adhesion and making the RPC more compact, thus improve RPC flexural strength [45,46,47]. However, when the content of WTSP exceeds 12%, the micro-aggregate ratio of RPC deviates from the optimal value. The large mass fraction of free WTSP particles and impurities affect the bonding between the quartz sand and the cementing material, resulting in a decrease in strength.

### 4.4. Analysis of Pore Structure Characteristics

**Figure 11:**
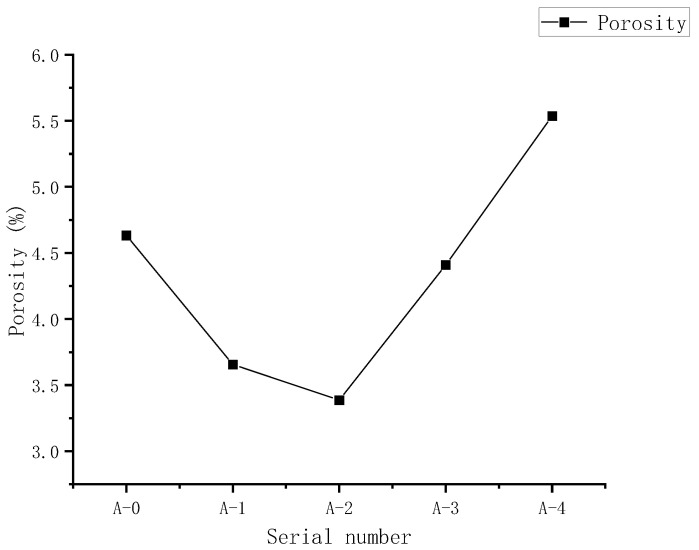
Twenty-eight-day porosity of RPC samples with different numbers.

**Figure 12:**
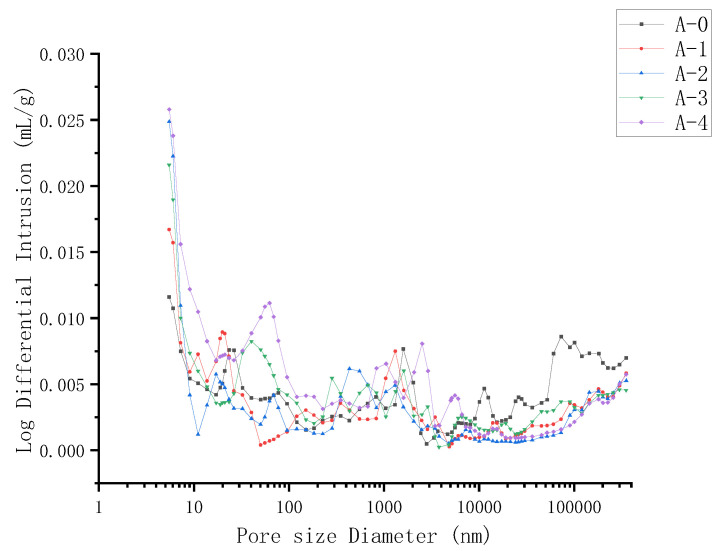
Twenty-eight-day pore size distribution of RPC samples with different numbers.

The pore structure characteristic of RPC samples cured for 28 days was also tested using the Mercury Intrusion Porosimetry (MIP). Figure 11 illustrates the cumulative porosity of sample A0–A4 cured for 28 days. As shown in Figure 11, the porosity of the sample A0–A3 is 4.6%, 3.7%, 3.4%, and 4.4%, when the WTSP content is 0%, 6%, 12%, and 18%, respectively. The addition of WTSP increases the number of fine particles in the RPC sample, filling the pores in the sample and forming a dense microstructure, reducing the porosity of the RPC sample. Figure 12 illustrates the pore size distribution of RPC samples A0–A4 cured for 28 days. When the WTSP content is 0%, 6%, 12%, and 18%, the average pore diameter of RPC is 41.43 nm, 27.04 nm, 24.30 nm, and 28.73 nm, respectively. From Figure 11 to Figure 12, it can be found that when the WTSP content is 12%, the larger diameter pores (>20 nm) in the RPC are significantly reduced, the concrete density is improved, and the porosity is reduced. However, when the WTSP content is more than 12%, the larger pores in RPC samples have already been filled where the nano-pores remain uninfluenced as the WTSP particles are more significant than the nano-pores. The increasing amount of WTSP will not further improve the micro-structure of the RPC sample [48,49]. Furthermore, the impurity content in the WTSP will cause a decrease in the compressive strength of the RPC, and the excess amount of WTSP may lead to the interfacial microcracks, resulting in a decrease in overall RPC UCS [41,50]. Hence, when the content of WTSP exceeds 12%, the addition of WTSP will not play a positive role in the mechanical properties development of RPC.

## 5. Conclusions

This research presents an experimental study of the effect of WTSP dosages on reactive powder concrete’s performance. Unconfined compressive strength and flexural strength were measured for RPC samples of all five mixes with three curing periods (3 days, 7 days and 28 days). Scanning electron microscopy and mercury intrusion porosimetry (MIP) testing was also carried out for selected RPC samples to identify the influence of WTSP content on the microstructural properties of RPC. Based on the results obtained, the following conclusions can be drawn from this study:

(1) The main crystalline minerals of WTSP are quartz, feldspar, and kaolinite, accounting for 81% of the total phase composition. The main chemical components are SiO_2_ and Al_2_O_3_, the content is as high as 77.92%, and the average particle size is 9.38 μm. The WTSP does not contain any corrosive substances. Through the 7-day and 28-day UCS tests, it was found that the WTSP has a certain activity for the hydration of cementitious material. The fine particles in WTSP could fill the large pores in RPC, leading to a dense microstructure. Hence, it is feasible to use solid waste WTSP to prepare reactive powder concrete.
(2) Under a constant water–binder ratio, when replacing the quartz sand with the same quality of the WTSP, the UCS of RPC gradually increases with the increase in curing age. With the increase in WTSP content, the UCS of RPC showed a downward trend and was found rather marginal. By adding 2% desulfurized gypsum to the A-4 sample, the compressive strength on day 3 and day 7 are significantly improved. However, the 28-day UCS remains unchanged. The RPC flexural strength shows a downward trend as the WTSP dosage increases. The flexural strength does not increase significantly by adding 2% desulfurized gypsum to the A-4 sample. RPC prepared with WTSP content within 12% can meet mechanical properties requirements [51]. At this content, the compressive strength of the RPC cube is 104.6 MPa, and the flexural strength is 12.0 MPa.
(3) The WTSP can react with tricalcium aluminate (C_3_A) and tetracalcium ferric aluminate (C_4_AF) to form hydrated calcium aluminate during the hydration process. The alkaline calcium carbonate in WTSP makes the structure of the micro-interface between hydration product tricalcium silicate (C_3_S) and CaCO_3_ denser. Moreover, Ca(OH)_2_ generates crystals on the surface of CaCO_3_, which makes the Ca(OH)_2_ grains refined, thereby improving the interfacial adhesion and making the RPC structure denser.
(4) The incorporated WTSP helps fill the larger diameter pores in RPC, hence improving the RPC’s pore structure and reducing the RPC’s porosity. When the content of WTSP is 12%, the total porosity of the sample is 0.0122 mL/g, which is reduced by 27.5% compared with reference sample A-0.

In this study, a WTSP–RPC preparation technology is proposed. Although it causes the loss of RPC mechanical properties, it still meets the requirements of RPC mechanical properties. It is a good choice to apply it to the RPC cover plate. In future work, through the laboratory test, the simplification of the WTSP–RPC curing process and the durability of WTSP–RPC will be studied.

## Figures and Tables

**Table 1:** Mixing ratio of wet WTSP instead of quartz sand (kg/m^3^).

	Cement	Silica Fume	Mineral Filler	Microbead Powder	Quartz Sand	Steel Fiber	WTSP	2% Desulfurization Gypsum	Defoamer	Water Reducing Agent	Water
A-0	650	180	120	200	1250	40	0	0	1	32	158
A-1	650	180	120	200	1175	40	75	0	1	40	158
A-2	650	180	120	200	1100	40	150	0	1	58	158
A-3	650	180	120	200	1025	40	225	0	1	77	158
A-4	650	180	120	200	1025	40	225	4.5	1	77	158

## Data Availability

Data are contained within the article.

## Appendix A

**Table A1:** Physical properties of Ordinary Portland Cement.

Specific Surface Area	Density	Standard Consistency	Soundness	Setting Time (min)		Flexural Strength (MPa)			Compressive Strength (MPa)		
m^2^/kg	g/cm^3^	%	Boiling Method	First Set	Final Set	3 days	7 days	28 days	3 days	7 days	28 days
355	3.12	25.4	qualified	99	158	6.4	7.3	9.3	27.0	37.8	53.2

**Table A2:** Chemical composition of Ordinary Portland Cement.

Element	SiO_2_	Al_2_O_3_	Fe_2_O_3_	CaO	MgO	SO_3_	Na_2_Oeq	f-CaO	Loss on Ignition	Cl^−^
Content (%)	20.58	4.97	3.76	63.57	2.29	2.00	0.53	0.75	1.40	0.026

**Table A3:** Physical and chemical properties of silica fume.

Detection Indicator	Total Alkali Content (%)	SiO_2_ Content (%)	Chlorine Content (%)	Moisture Content (%)	Ignition Loss (%)	Water Demand Ratio (%)	Specific Surface Area m^2^/kg	7 days Activity Index	Radioactivity
Test results	0.88	94.61	0.016	1.4	1.26	112	18,648	120	none

**Table A4:** Physical and chemical properties of microbeads.

Detection Indicator	45 μm Sieve Residue (%)	Water Demand Ratio (%)	Ignition Loss (%)	SO_3_ Content (%)	f-CaO (%)	Moisture Content (%)
Test results	12.5	98	2.5	0.1	0	0.1

**Table A5:** Physical and chemical properties of mineral powder.

Detection Indicator	Density (g/cm^3^)	Specific Surface Area (m^2^/kg)	Ignition Loss (%)	7 days Activity Index	28 days Activity Index	Flow Ratio (%)	Moisture Content (%)
Test results	2.9	455	2.3	78	96	100	0.3

**Table A6:** Chemical composition analysis of quartz sand.

Element	SiO_2_	Al_2_O_3_	Fe_2_O_3_	K_2_O	Na_2_O
Content (%)	98.21	0.83	0.03	0.02	0.02

**Table A7:** Properties of WTSP.

Performance	Density (g/cm^3^)	Methylene Blue Value	Surface Area/Volume (cm^2^/cm^3^)	The Average Particle Size (μm)	7 days Activity Index (%)	28 daysActivity Index (%)	Water Demand Ratio (%)	pH
Test results	2.561	9	14,225	9.38	55.3	59.3	117.2	8.95

**Table A8:** Phase analysis of WTSP.

Phase	Quartz	Feldspar	Calcite	Kaolinite	Chlorite	Lllite	Montmorillonite	Pyrite
Content (%)	20.6	45.9	5.9	14.5	1.1	4	7.7	0.2

**Table A9:** Chemical composition of WTSP.

Element	CaO	MgO	SiO_2_	Al_2_O_3_	Fe_2_O_3_	FeO	TiO_2_	Na_2_O	MnO	K_2_O	P_2_O_5_	H_2_O^+^	SO_3_
Content (%)	3.20	0.44	61.52	16.40	4.28	1.95	0.49	2.28	0.40	4.91	0.06	2.51	<0.1

**Table A10:** Corrosion analysis of WTSP.

Test Items	pH	Ca^2+^	Mg^2+^	Cl^−^	CO_3_^2−^	HCO_3_^−^	SO_4_^2−^
Content (mg/kg)	8.95	0.081	0.012	0.054	0.000	0.308	0.078
